# Transforming Hematology Care in Africa: Strategies for Enhanced Diagnosis and Treatment

**DOI:** 10.1002/hsr2.72396

**Published:** 2026-04-15

**Authors:** Emmanuel Ifeanyi Obeagu

**Affiliations:** ^1^ Division of Haematology, Department of Biomedical and Laboratory Science Africa University Zimbabwe; ^2^ Department of Molecular Medicine and Haematology, Faculty of Health Sciences University of the Witwatersrand Johannesburg South Africa

**Keywords:** Africa, diagnostics, healthcare systems, hematology, treatment

## Abstract

**Background:**

Hematologic disorders contribute significantly to morbidity and mortality across Africa. Challenges include delayed diagnosis, limited access to essential therapies, workforce shortages, and fragmented blood transfusion services. Although global advances in diagnostics and therapeutics exist, their translation into African contexts remains inconsistent.

**Objective:**

To provide a comprehensive narrative review of the current state of hematology care in Africa, highlighting diagnostic and treatment gaps, country‐level experiences, and evidence‐informed strategies for improving patient outcomes.

**Methods:**

A structured literature search was conducted using PubMed, Scopus, and Web of Science for publications from 2018 to 2025. Search terms included “hematology Africa,” “sickle cell disease,” “blood disorders,” “diagnostics,” “blood transfusion,” and “hematologic malignancies.” Studies were selected based on relevance to diagnostic strategies, treatment interventions, health system strengthening, and country‐level programs. Thematic synthesis was used to identify recurrent challenges and strategic interventions.

**Results:**

Key challenges in hematology care include limited access to advanced diagnostics, insufficient laboratory and clinical workforce, inconsistent availability of essential medicines, and weak blood transfusion infrastructure. Country‐level examples illustrate both successes and gaps: Rwanda has implemented neonatal sickle cell screening and drone‐assisted blood delivery; Nigeria has regional sickle cell centers, but uneven coverage; Uganda and Kenya have improved diagnostic capacity through regional referral laboratories and point‐of‐care testing; Ghana has expanded access to targeted therapies for chronic myeloid leukemia via national health insurance programs. Strategic interventions include decentralizing diagnostics, expanding point‐of‐care testing, strengthening blood services, enhancing workforce training and CME, leveraging digital health and teleconsultation, and promoting regional centers of excellence. Collaborative initiatives and context‐adapted policies are critical for sustainability.

**Conclusion:**

Transforming hematology care in Africa requires integrated, evidence‐informed strategies combining technological innovation with health system strengthening. Country‐specific experiences highlight actionable pathways, while persistent gaps underscore the need for scalable, equitable, and sustainable approaches. Coordinated efforts in diagnostics, therapeutics, workforce development, and service delivery can improve outcomes for patients with hematologic disorders across the continent.

**Abbreviations**
AIDS: acquired immune deficiency syndrome
AJOL: African journals online
CDC: centers for disease control and prevention
DNA: deoxyribonucleic acid
HIV: human immunodeficiency virus
LMICs: low‐ and middle‐income countries
NCDs: non‐communicable diseases
PCR: polymerase chain reaction
RBC: red blood cell
SCD: sickle cell disease
SSA: sub‐Saharan Africa
WBC: white blood cell
WHO: World Health Organization

## 1 Introduction

Hematology, the branch of medicine concerned with the study and treatment of blood disorders, is a vital component of any health system. In Africa, hematologic conditions such as sickle cell disease (SCD), anemia, thalassemia, hemophilia, and hematologic malignancies represent a significant burden on individuals, families, and healthcare infrastructures. These diseases are associated with high morbidity and mortality, particularly in regions where access to specialized care is limited or nonexistent [1, 2, 3]. The burden of hematologic diseases in Africa is exacerbated by various social, economic, and health system constraints. Sickle cell disease, for instance, is one of the most prevalent inherited disorders in sub‐Saharan Africa, with an estimated 75% of the global SCD births occurring on the continent. Meanwhile, iron deficiency anemia remains a leading cause of disability in children and pregnant women. Despite the urgency of addressing these conditions, many patients remain undiagnosed or inadequately managed due to fragmented and underfunded health services [4, 5, 6]. One of the critical barriers to improved hematology care in Africa is the lack of diagnostic infrastructure. Many health facilities across the continent lack the equipment, reagents, and skilled personnel required to perform even basic hematologic tests such as complete blood counts (CBCs), blood film analysis, or hemoglobin electrophoresis. In rural and peri‐urban settings, patients often rely on clinical suspicion alone, leading to misdiagnosis or delayed intervention [7, 8].

In addition to diagnostic limitations, access to effective and affordable treatment remains a major challenge. Essential medicines such as hydroxyurea, iron supplements, folic acid, and clotting factor concentrates are either unavailable or unaffordable for a large segment of the population. Where treatment is available, it is often concentrated in urban tertiary hospitals, leaving rural populations underserved. Furthermore, the absence of standardized treatment protocols and national guidelines further contributes to inconsistent and suboptimal care [9, 10, 11]. Another pressing issue is the shortage of trained hematologists, laboratory scientists, and allied health professionals with expertise in hematology. Many African countries have only a handful of specialists, who are often overburdened and concentrated in major cities. This has led to a cycle of inadequate training and mentorship for the next generation of healthcare providers, further weakening the capacity of health systems to manage hematologic conditions [12, 13].

## 2 Aim

This review aims to critically explore the current state of hematology care in Africa, highlighting the prevailing diagnostic and treatment challenges while proposing strategic interventions to improve outcomes.

## 3 Review Methods

To provide a comprehensive and context‐specific analysis of hematology care in Africa, this narrative review employed a structured approach to the identification, selection, and synthesis of relevant literature. A broad search was conducted across multiple academic databases, including PubMed, Scopus, Google Scholar, Web of Science, and African Journals Online (AJOL), covering publications from 2000 to 2024. The search strategy incorporated a combination of key terms such as “hematologic diseases in Africa,” “blood disorders,” “diagnostic challenges,” “hematology infrastructure,” “treatment gaps,” “health policy,” and “capacity building,” along with country‐specific and regional modifiers.

Inclusion criteria were guided by relevance to the review's objectives, with emphasis placed on peer‐reviewed articles, institutional reports, government health bulletins, and policy briefs that addressed hematology‐related issues in African settings. Studies that focused on both communicable and non‐communicable hematologic conditions—including anemia, sickle cell disease, hemophilia, leukemia, and lymphomas—were considered. Grey literature, particularly reports from WHO, Africa CDC, and international hematology organizations, was also reviewed to supplement academic findings with real‐world insights and programmatic evidence.

## 4 Burden of Hematologic Diseases in Africa

Across the vast and diverse landscape of Africa, hematologic diseases continue to pose a formidable challenge to public health and clinical care. From the bustling urban centers to the most remote rural communities, these disorders silently affect millions of lives, often going undetected until complications arise. Among the most prominent is sickle cell disease (SCD)—a genetic blood disorder that has become almost synonymous with African hematology. It is estimated that over 300,000 infants are born globally with SCD each year, and the majority of these births occur in sub‐Saharan Africa. Without early diagnosis and comprehensive care, most of these children do not survive beyond their fifth birthday [14, 15, 16]. But SCD is only one facet of the continent's hematologic burden. Iron deficiency anemia remains rampant, particularly among women of reproductive age, pregnant women, and young children, often resulting from nutritional deficiencies, chronic infections, and parasitic infestations. The implications of untreated anemia are far‐reaching, contributing to increased maternal and infant mortality, cognitive impairments, and decreased productivity. In areas plagued by malaria, HIV, and tuberculosis, anemia is further compounded, creating a vicious cycle that weighs heavily on both individuals and already overstretched health systems [17, 18, 19, 20].

In parallel, inherited bleeding disorders such as hemophilia and thalassemia persist in the shadows, largely underdiagnosed and frequently misunderstood. Due to limited awareness and diagnostic capabilities, many patients live with repeated bleeding episodes or chronic complications, often mistaken for other conditions. Leukemias and lymphomas, which are among the most common hematologic malignancies, are increasingly being recognized across African oncology units, yet access to bone marrow biopsies, immunophenotyping, and cytogenetics remains rare in many parts of the continent [21, 22]. The impact of these diseases is not just clinical—it is deeply social and economic. Families face the constant emotional and financial toll of managing chronic conditions with few resources. Children miss school due to recurrent pain crises or hospital visits, while adults may lose jobs or livelihoods because of untreated symptoms or long hospitalizations. Moreover, cultural stigma and lack of awareness often discourage affected individuals from seeking timely care, reinforcing cycles of neglect and poor outcomes [23, 24].

### 4.1 Challenges in Diagnosis and Treatment of Hematologic Disorders in Africa

The diagnosis and treatment of hematologic disorders in Africa are constrained by interrelated structural, technical, and clinical challenges that vary substantially across countries and health system levels. While tertiary centers in a few urban settings have developed specialized hematology and oncology services, large segments of the population continue to rely on under‐resourced primary and district‐level facilities, where diagnostic capacity is limited, and referral pathways are fragmented. These disparities contribute to delayed diagnosis, advanced disease at presentation, and suboptimal treatment outcomes across both benign and malignant hematologic conditions [25]. Diagnostic constraints remain a foundational bottleneck. In many settings, hematologic evaluation is still largely dependent on basic microscopy and limited full blood count availability, with inconsistent access to confirmatory testing such as hemoglobin electrophoresis, flow cytometry, cytogenetics, and molecular diagnostics. This diagnostic gap contributes to frequent misclassification of conditions such as sickle cell disease, leukemias, and lymphomas, particularly outside urban referral centers. For example, while neonatal screening for sickle cell disease has been piloted or implemented in selected countries, coverage remains uneven, and many individuals are diagnosed only after recurrent complications in childhood or adolescence. Similarly, access to molecular diagnostics for conditions such as chronic myeloid leukemia remains concentrated in a small number of regional laboratories, resulting in delayed initiation of targeted therapies and limited capacity for treatment monitoring [26, 27].

Therapeutic limitations are closely linked to diagnostic gaps and are compounded by constraints in drug availability, affordability, and supportive care infrastructure. Essential medicines for anemia management, bleeding disorders, and hematologic malignancies are not consistently available in many public‐sector facilities, and treatment interruptions due to stock‐outs are frequently reported. Blood transfusion services, which are central to the management of severe anemia and hemoglobinopathies, remain vulnerable to shortages, variable screening capacity, and limited component separation in several countries. Even where effective therapies exist, late‐stage presentation often reduces the likelihood of favorable outcomes, particularly for aggressive malignancies that require timely, protocol‐driven treatment [28]. Health system and workforce challenges further shape diagnostic and treatment outcomes. The density of trained hematologists, pathologists, laboratory scientists, and oncology nurses remains low in many countries, contributing to diagnostic delays and variability in treatment quality. Task‐shifting strategies and protocol‐based care have expanded service delivery in some contexts, but uneven training, high staff turnover, and limited continuing professional development constrain sustainability. Referral pathways between primary, secondary, and tertiary care are often poorly coordinated, leading to loss to follow‐up and delays in treatment initiation [29].

Geographic and socioeconomic barriers also influence access to care. Patients in rural and peri‐urban areas face long travel distances to referral centers, indirect costs related to transportation and accommodation, and out‐of‐pocket expenditures for diagnostics and medicines. These barriers contribute to delayed health‐seeking behavior and treatment discontinuation. While innovative service delivery models, such as decentralized diagnostics and mobile outreach, have shown promise in selected settings, their scale‐up remains uneven and dependent on external funding and local health system capacity (Table 1) [30].

**Table 1:** Challenges in diagnosis and treatment of hematologic disorders in Africa.

Challenge domain	Specific barriers	Illustrative country examples	Implications for patient outcomes
Diagnostic limitations	Limited access to advanced diagnostics (flow cytometry, molecular testing, cytogenetics); reliance on microscopy; uneven neonatal screening	Rwanda: Neonatal sickle cell screening pilot improved early detection, but coverage remains limited outside urban areas. Uganda/Kenya: Regional labs expanded molecular testing for leukemia and lymphoma.	Delayed or missed diagnosis; misclassification of hematologic conditions; delayed initiation of targeted therapies
Therapeutic access	Inconsistent availability of essential medicines (hydroxyurea, factor concentrates, chemotherapy); stock‐outs; high out‐of‐pocket costs	Ghana: National health insurance enabled access to tyrosine kinase inhibitors for CML; Nigeria: Regional sickle cell centers provide hydroxyurea, but limited in rural areas	Interruptions in treatment, progression of disease, increased morbidity, and mortality
Blood transfusion services	Limited voluntary donations, component preparation, and hemovigilance; geographic barriers	Rwanda: Drone‐assisted blood delivery reduced transfusion delays; Nigeria/Kenya: Mobile donation campaigns increased supply, but rural coverage remains uneven	Delayed transfusions; inadequate management of severe anemia and hemoglobinopathies
Workforce and training	Shortage of hematologists, pathologists, laboratory scientists, and oncology nurses; uneven distribution	Uganda/Kenya: Training programs and international partnerships improved lab and clinical capacity; retention challenges persist	Diagnostic delays, variability in care quality, and limited access to specialized interventions
Geographic and socioeconomic barriers	Long travel distances, high transport costs, and rural health facility limitations	Nigeria/Ghana: Patients in remote areas face delayed presentation; limited local referral capacity	Late‐stage disease presentation; treatment discontinuation; inequitable outcomes
Health system coordination	Fragmented referral pathways; limited integration of diagnostics, treatment, and follow‐up	Kenya/Rwanda: Teleconsultation and digital health platforms improved continuity of care; scaling remains limited	Loss to follow‐up; reduced treatment adherence; inconsistent monitoring and outcome tracking

### 4.2 Strategic Interventions for Transforming Hematology Care in Africa

Transforming hematology care in Africa requires coordinated, multi‐level interventions that align technological innovation with health system strengthening, workforce development, and sustainable financing mechanisms. Rather than replicating high‐income country models, effective strategies must be adapted to local epidemiology, resource constraints, and existing care pathways. Emerging evidence from country‐level initiatives and regional collaborations highlights several priority intervention domains [31]. Strengthening tiered diagnostic networks is a foundational intervention. Decentralized point‐of‐care testing for anemia, coagulation disorders, and hemoglobinopathies can improve early case identification at primary and district levels, while regional referral laboratories provide access to flow cytometry, cytogenetics, and molecular diagnostics for definitive classification of hematologic malignancies. For example, pilot neonatal screening programs for sickle cell disease in selected countries have demonstrated improved early diagnosis and linkage to care, although coverage and long‐term sustainability remain limited by funding and laboratory capacity. Investments in laboratory quality management systems, sample referral networks, and digital connectivity are necessary to ensure diagnostic reliability and continuity across tiers of care [6, 32].

Expanding access to essential therapies and supportive care is equally critical. Alignment of national essential medicines lists with evidence‐based hematology guidelines can improve the availability of first‐line therapies for anemia, bleeding disorders, and hematologic malignancies. Regional pooled procurement mechanisms and public–private partnerships offer opportunities to reduce costs and stabilize supply chains for chemotherapeutic agents, factor concentrates, and supportive care medicines. Strengthening blood transfusion services—including voluntary donation programs, component separation, and hemovigilance—remains central to managing severe anemia and transfusion‐dependent conditions. While advanced therapies such as stem cell transplantation are available in only a limited number of centers, strategic development of regional centers of excellence can incrementally expand access, provided that referral pathways and long‐term follow‐up systems are strengthened [33, 34]. Workforce development and continuing medical education (CME) represent critical enablers of sustainable transformation. Expanding local training programs for hematologists, pathologists, laboratory scientists, and oncology nurses can reduce dependence on external expertise. Task‐shifting and standardized treatment protocols have extended service delivery in some settings, but their effectiveness depends on structured supervision and ongoing training. Virtual CME initiatives and international mentorship networks have improved access to specialist input in resource‐limited settings, although challenges related to digital infrastructure, time zone coordination, and continuity of mentorship remain. Systematic evaluation of these educational interventions is needed to better quantify their impact on clinical practice and patient outcomes [35].

Leveraging digital health and innovative service delivery models offers additional opportunities to bridge geographic and infrastructure gaps. Tele‐hematology platforms can facilitate remote diagnostic review and multidisciplinary case discussions, while electronic medical records and laboratory information systems support continuity of care and outcome tracking. Innovative logistics solutions, such as drone‐assisted delivery of blood products in selected settings, demonstrate the potential to reduce transfusion delays in hard‐to‐reach areas. However, scalability, regulatory frameworks, cost‐effectiveness, and long‐term sustainability require careful evaluation before widespread adoption [36]. Embedding implementation science and health system integration is essential to translate pilot successes into durable system‐wide improvements. Interventions must be accompanied by robust monitoring and evaluation frameworks, context‐specific adaptation, and policy alignment with national health strategies and universal health coverage goals. Equitable research partnerships and regional centers of excellence can support locally relevant evidence generation, clinical trials, and capacity building. Importantly, strategies should be designed with sustainability in mind, minimizing dependence on short‐term external funding and ensuring alignment with domestic financing and governance structures (Table 2) [37].

**Table 2:** Strategic interventions for transforming hematology care in Africa.

Intervention domain	Key strategies	Country‐level examples	Observed/anticipated impact	Limitations/considerations
Decentralized diagnostics and POC testing	Point‐of‐care (POC) testing for anemia, sickle cell disease, and coagulation disorders; tiered lab networks with referral hubs	Rwanda: Neonatal sickle cell screening programs; Kenya: POC testing integrated in county hospitals	Early diagnosis, improved linkage to care, and reduced complications	Coverage still limited in rural areas; requires quality assurance and training
Therapeutic access and essential medicines	Alignment of essential medicines lists; regional pooled procurement; risk‐adapted treatment protocols	Ghana: Access to tyrosine kinase inhibitors for CML under national health insurance; Nigeria: Hydroxyurea therapy in regional sickle cell centers	Improved adherence, reduced disease progression, expanded treatment access	Supply chain fragility; cost constraints; urban–rural disparities
Blood transfusion services	Voluntary donor programs, component separation, hemovigilance, mobile donation campaigns	Rwanda: Drone‐assisted blood delivery; Kenya: Regional blood banks and mobile donation drives	Reduced transfusion delays, improved management of anemia and hemoglobinopathies	Infrastructure‐intensive; scalability challenges in remote areas
Workforce development and CME	Specialist training pipelines, task‐shifting, mentorship, and virtual CME	Uganda/Kenya: Lab scientist and hematologist training supported by international partnerships; ASH and iCML virtual CME programs	Increased diagnostic and treatment capacity, improved quality of care	Retention challenges; variable digital infrastructure; ongoing supervision needed
Digital health and teleconsultation	Telepathology, virtual case review, electronic medical records, digital referral networks	Kenya/Rwanda: Tele‐hematology platforms linking district hospitals with tertiary centers	Improved continuity of care, enhanced decision support, and reduced diagnostic delays	Requires reliable connectivity; sustainability depends on funding and integration into health systems
Regional centers of excellence and collaborative networks	Integrated care centers, research partnerships, shared infrastructure	Ghana/Nigeria: CML and sickle cell collaborative networks; Rwanda: National centers supporting training and implementation research	Consolidated expertise, improved patient outcomes, and evidence generation	High initial investment; may not be immediately accessible to rural populations
Implementation science and policy integration	Monitoring and evaluation frameworks, context‐adapted protocols, policy alignment with UHC goals	Multi‐country initiatives guided by WHO and regional health authorities	Sustainable scale‐up of interventions, improved health system responsiveness	Requires sustained political and financial commitment; coordination across multiple stakeholders

## 5 Country‐Level Experiences in Hematology Care Across Africa

Hematology care across Africa reflects considerable heterogeneity, shaped by differences in healthcare infrastructure, economic capacity, disease burden, and policy priorities. To illustrate this diversity, selected country‐level experiences from different regions of the continent—including West, East, Southern, and North Africa—are highlighted. These examples are not intended to represent all African countries but rather to provide illustrative insights into how varying health system structures and resource contexts influence the delivery of hematology services [38, 39].

### 5.1 East Africa

In Kenya and Tanzania, hematology services are expanding alongside broader investments in healthcare infrastructure and laboratory medicine. National referral hospitals and university teaching hospitals increasingly provide specialized hematology services, including bone marrow examination, flow cytometry in selected centers, and improved diagnostic capabilities for hematological malignancies. However, disparities between urban tertiary centers and peripheral health facilities remain significant. Malaria‐associated anemia and nutritional deficiencies continue to contribute substantially to hematologic morbidity in this region. Collaborative research initiatives and partnerships with international institutions have played an important role in strengthening diagnostic capacity and supporting clinical training in hematology and laboratory medicine [40, 41].

### 5.2 West Africa

In West Africa, countries such as Nigeria and Ghana face a substantial burden of hematological disorders, particularly SCD, which represents one of the most prevalent inherited blood disorders in the region. Nigeria accounts for the largest number of global SCD births annually, creating a significant demand for diagnostic services, comprehensive care programs, and transfusion support. While tertiary institutions in major cities have established hematology units equipped with automated hematology analyzers and some advanced diagnostic technologies, many rural healthcare facilities still rely on basic laboratory methods. Efforts to strengthen newborn screening programs, expand hydroxyurea therapy, and improve access to safe blood transfusion services are gradually transforming SCD management in these countries. Ghana has also made progress through national public health initiatives, including the development of newborn screening programs and specialized SCD clinics, which serve as models for regional hematology care improvement [42].

### 5.3 Southern Africa

Southern African countries such as South Africa and Zimbabwe demonstrate a mixture of advanced capabilities and resource constraints. South Africa possesses one of the most developed healthcare and laboratory systems on the continent, with access to sophisticated diagnostic platforms including cytogenetics, molecular diagnostics, and hematopoietic stem cell transplantation services. These capabilities have facilitated improved diagnosis and management of hematologic malignancies and inherited blood disorders. Conversely, countries such as Zimbabwe continue to face resource limitations that affect diagnostic infrastructure and access to specialized therapies. Nonetheless, academic institutions and national health programs are increasingly investing in laboratory strengthening, training of hematologists, and improved blood transfusion services to address the growing burden of hematological diseases [43, 44].

### 5.4 North Africa

North African countries—including Egypt, Morocco, and Tunisia—present a somewhat different healthcare landscape compared with many sub‐Saharan African regions. These countries generally have more established tertiary healthcare institutions and relatively stronger diagnostic infrastructures, including access to molecular diagnostics and specialized hematology services. Egypt, for example, has developed several specialized centers for the management of hemoglobinopathies and hematological malignancies, supported by active research programs and expanding genomic medicine initiatives. Morocco and Tunisia have also made progress in strengthening blood transfusion services and implementing national strategies for the management of inherited blood disorders such as thalassemia. Despite these advances, North African countries still face challenges related to increasing demand for advanced therapies, equitable access to specialized care across urban and rural areas, and the rising burden of hematologic cancers [45, 46].

### 5.5 Cross‐Regional Lessons

Across the continent, several common themes emerge from these country‐level experiences. First, strengthening laboratory diagnostic capacity remains a central priority for improving hematology care. Second, the training and retention of specialized healthcare professionals, including hematologists and laboratory scientists, is critical for sustainable service delivery. Third, robust blood transfusion systems are essential for managing many hematologic conditions, particularly in regions with a high prevalence of anemia and hemoglobinopathies. Finally, expanding research collaborations and regional networks can facilitate knowledge exchange, promote innovation, and support the development of context‐specific solutions to hematological health challenges in Africa (Table 3) [40, 47].

**Table 3:** Country‐level experiences in hematology care across Africa.

Region	Country	Key hematological burden	Current strengths in hematology care	Major challenges	Notable initiatives/progress
West Africa	Nigeria	High prevalence of sickle cell disease (SCD), anemia, hematological malignancies	Presence of tertiary hematology centers, expanding laboratory diagnostics, and specialized clinics for SCD	Limited rural diagnostic access, inadequate blood supply, high treatment costs	Newborn screening advocacy, expansion of hydroxyurea therapy, national SCD control programs
West Africa	Ghana	SCD, nutritional anemia, transfusion‐related conditions	Established newborn screening programs, dedicated SCD clinics	Resource disparities between urban and rural healthcare facilities	National SCD programs and strengthened public health interventions
East Africa	Kenya	Malaria‐related anemia, hematological malignancies, hemoglobinopathies	Growing laboratory infrastructure, university teaching hospitals providing specialized hematology services	Limited access to advanced diagnostics such as cytogenetics and molecular testing	International collaborations supporting research and training
East Africa	Tanzania	Severe anemia, hemoglobinopathies, hematologic malignancies	National referral hospitals with expanding hematology units	Workforce shortages, limited diagnostic technologies in peripheral facilities	Capacity building through global health partnerships
Southern Africa	South Africa	Leukemia, lymphoma, SCD, HIV‐associated hematologic disorders	Advanced diagnostic technologies, cytogenetics, molecular diagnostics, and stem cell transplantation	Healthcare disparities between public and private sectors	Established hematology research centers and national blood services
Southern Africa	Zimbabwe	Anemia, SCD, hematologic malignancies	Academic institutions supporting hematology training, improving laboratory services	Limited funding, shortages of specialized equipment, and reagents	Ongoing laboratory strengthening and transfusion service improvements
North Africa	Egypt	Thalassemia, SCD, hematological malignancies	Specialized hematology centers, molecular diagnostics, and genomic research initiatives	Increasing demand for advanced therapies and equitable access	National programs for hemoglobinopathy management
North Africa	Morocco	Thalassemia, leukemia, lymphoma	Expanding diagnostic laboratories and transfusion services	Unequal access between urban and rural areas	National initiatives to strengthen blood safety and hematology care
North Africa	Tunisia	Hemoglobinopathies, hematologic cancers	Strong tertiary healthcare institutions and specialist services	Rising treatment costs and access to advanced therapeutics	Government‐supported healthcare programs and clinical research

## 6 Conclusion

The burden of hematologic diseases in Africa presents a complex and pressing public health challenge, exacerbated by diagnostic limitations, treatment disparities, and systemic constraints. However, with coordinated and strategic interventions, these challenges are not insurmountable. Strengthening diagnostic infrastructure, expanding the hematology workforce, enhancing public health policies, and promoting research are essential to establishing a more equitable and effective hematology care system across the continent. Moreover, community engagement and culturally sensitive education can dispel misconceptions and encourage early intervention, while international partnerships can bridge resource gaps and facilitate technology transfer. The integration of digital tools and telemedicine adds a forward‐looking dimension, offering scalable solutions to longstanding access barriers.

## Author Contributions

**Emmanuel Ifeanyi Obeagu:** conceptualization, writing – original draft, methodology, validation, visualization, writing – review and editing, and supervision.

## Funding

The author has nothing to report.

## Conflicts of Interest

The author declares no conflicts of interest.

## Transparency Statement

The lead author, Emmanuel Ifeanyi Obeagu, affirms that this manuscript is an honest, accurate, and transparent account of the study being reported; that no important aspects of the study have been omitted; and that any discrepancies from the study as planned (and, if relevant, registered) have been explained.

## Data Availability

The author has nothing to report.
